# Archaeology and ichnology at Gombore II-2, Melka Kunture, Ethiopia: everyday life of a mixed-age hominin group 700,000 years ago

**DOI:** 10.1038/s41598-018-21158-7

**Published:** 2018-02-12

**Authors:** Flavio Altamura, Matthew R. Bennett, Kristiaan D’Août, Sabine Gaudzinski-Windheuser, Rita T. Melis, Sally C. Reynolds, Margherita Mussi

**Affiliations:** 1 Dipartimento di Scienze dell’Antichità, Università di Roma Sapienza, Piazzale A. Moro 5, 00185 Rome, Italy; 2 Italian Archaeological Mission at Melka Kunture and Balchit (Ethiopia), Rome, Italy; 3 Institute for Studies in Landscapes and Human Evolution, Bournemouth University, Poole, BH12 5BB UK; 4 Department of Musculoskeletal Biology, Institute of Ageing and Chronic Disease, University of Liverpool, Liverpool, L7 8TX UK; 5 MONREPOS Archaeological Research Centre and Museum for Human Behavioural Evolution and Institute of Ancient Studies, Johannes Gutenberg–University Mainz, Schloss Monrepos, 56567 Neuwied, Germany; 6 Dipartimento di Scienze Chimiche e Geologiche, Università di Cagliari, Via Trentino 51, 009127 Cagliari, Italy

## Abstract

We report the occurrence at 0.7 million years (Ma) of an ichnological assemblage at Gombore II-2, which is one of several archaeological sites at Melka Kunture in the upper Awash Valley of Ethiopia, 2000 m asl. Adults and children *potentially* as young as 12 months old left tracks in a silty substrate on the shore of a body of water where ungulates, as well as other mammals and birds, congregated. Furthermore, the same layers contain a rich archaeological and palaeontological record, confirming that knapping was taking place *in situ* and that stone tools were used for butchering hippo carcasses at the site. The site gives direct information on hominin landscape use at 0.7 Ma and may provide fresh perspective on the childhood of our ancestors.

## Introduction

**Figure 1:**
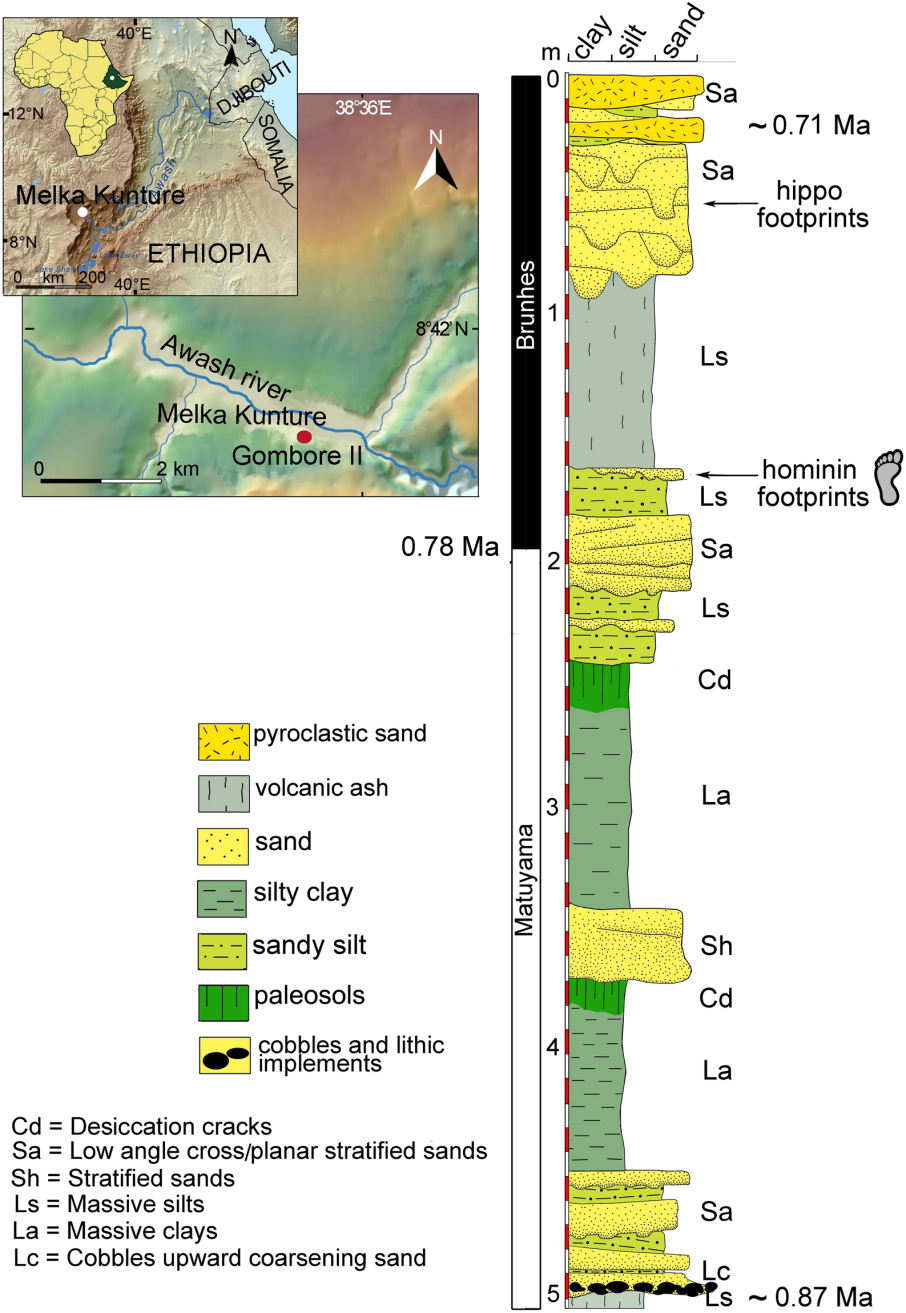
Gombore II-2: location and stratigraphy (map of Ethiopia modified from https://commons.wikimedia.org/w/index.php?curid = 11107194 (CC BY-SA 3.0; https://creativecommons.org/licenses/by-sa/3.0/deed.en); DEM of the Awash River created using unpublished maps in the archives of the Italian Archeological Mission at Melka Kunture and Balchit).

Fossil footprints provide insight into palaeoenvironments and palaeoecology and allow more detailed reconstruction of the landscapes where hominins lived. Footprints can also open a window on human behaviour and interaction with associated animals, thereby providing a snapshot of past life^1^. We report on a tracksite at Gombore II-2 (Melka Kunture) in the Upper Awash Valley of Ethiopia (Fig. 1) where a trampled surface, including hominin tracks, was preserved by an ash-flow surge, dated to 0.7 Ma^2^. On the basis of chronology, and in the light of hominin remains at Melka Kunture itself^3^, the track-maker is assumed to be *Homo heidelbergensis*. Tracks of mammals, ranging from small-sized gazelles to hippos and birds were also imprinted on the soft ground. The tracksite is directly associated with a rich archaeological record, including stone tools, fossil fauna and evidence of butchery. This allows us to establish an accurate reconstruction of the local environment and of hominin activities undertaken there.

## History of Excavations, Stratigraphic Sequence and Geochronology

In the area of Melka Kunture (N 8°42.284′; E 38°36.098′) a French Mission led by Jean Chavaillon started the research in 1965. From 1999 onwards the responsibility for investigations has been held by the Italian Archaeological Mission at Melka Kunture and Balchit and is now directed by Margherita Mussi of Sapienza University of Rome^4,5^. Tens of sites, often multi-layered, have been excavated over the past 50 years in the gullies and valley tributaries of the Awash, providing rich and complex archaeological, palaeontological, palaeoanthropological and palaeoenvironmental evidence^4^.

Gombore II-2 is in the upper part of Gombore gully on the right bank of the Awash River (Fig. 1). In 1974, Acheulean implements were first recovered at Gombore II-2, leading to systematic surface collection and to test excavations. Fieldwork resumed in 1993 and in 1995, with excavations totalling 26 m^2^. Scientific interest at the time was focused on the association of hippopotamus bones with lithics, suggesting that it was a butchering site^4^. From 2013 to 2015, some of the authors excavated over ~35 m^2^ more (Supplementary Fig. S1), discovering a stratigraphic sequence more complex than previously recorded^5^. They also found cross-cutting footprints of hominins and other animals suggesting repeated congregation at this site over a short period of time.

Between 1.8 and 0.2 Ma the palaeo-Awash River meandered in a half-graben that accumulated fluvial and fluvial-lacustrine sediments. The deposits were often interbedded with volcanic products, mostly distal ashes erupted by volcanic centers located a few tens of kilometers away^2,4^. Archaeological horizons usually occur within the fluvial-lacustrine deposits. In the Gombore gully (Fig. 1), the footprint-bearing units are bracketed by volcanic sediments dated by ^40^Ar/^39^Ar to 0.875 ± 0.010 Ma and 0.709 ± 0.013 Ma, respectively^2^. Further chronological constraint is provided at ~0.78 Ma by the Matuyama/Brunhes magnetostratigraphic boundary identified by Tamrat and colleagues^6^. The location of this has been recently reassessed within the stratigraphic sequence^5^ and lies ~0.4 m below the tracks reported here (Fig. 1). Between the dated volcanic deposits, we recorded a ~5.0 m-thick stratigraphic sequence (Fig. 1). At the base is an extensive Middle Acheulean layer^7^, overlying the 0.875 Ma volcanic sediments and formed by a dense accumulation of cobbles, lithic and faunal remains, extending over an area of ~1000 m^2^. In 1974 and 1976 two human cranial fragments were recovered from this layer. They were recently referred to an early form of *H. heidelbergensis*^3^.

The Acheulean layer is covered by ~0.50 m-thick sandy and silty channel deposits and ~2.0 m massive silty clay interbedded by sands, interpreted as overbank flood deposits, with palaeosol development. Stratified sands interbedded with silty-sandy sediments with fossil and lithic remains accumulated above the uppermost palaeosol, suggesting recurrent anthropogenic activity^5^. The footprints occur within the uppermost sandy silt layer (0.2 m thick). Sands filled the tracks. In the central-eastern part of the excavated area, sand lenses accumulated to a maximum thickness of 0.1 m over ~20 m^2^. To the west, the sands are residual, and the trampled sandy silt layer outcropped over further ~15 m^2^ (Supplementary Fig. S1). The ichnosurface was affected neither by pedogenetic processes nor by desiccation cracks, suggesting that it was not exposed for a substantial length of time before ~1 m of volcanic ash sealed the palaeo-landscape. The sequence ends with volcanic sands with more tracks, printed exclusively by hippos settling again after the volcanic event, and with the dated pyroclastic deposit (0.709 Ma)^8^. The fine-grained sediments of this upper part of the Pleistocene sequence (i.e. interbedded sands, silts and clayey volcanic ashes) were plastic and humid enough to allow the production and preservation of fossil footprints^9–11^.

## Description of the Footprint Surface: Ichnology

**Figure 2:**
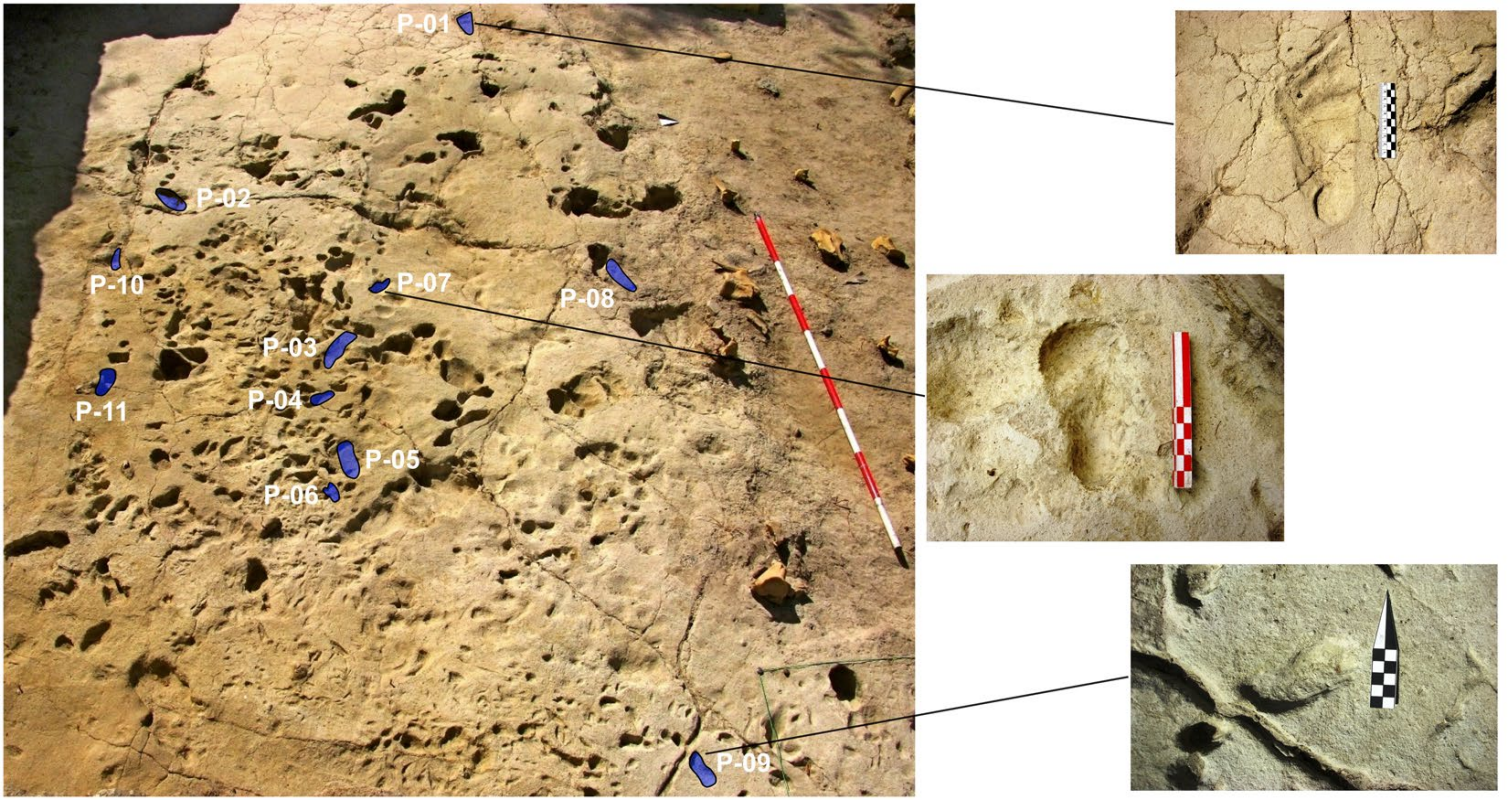
Overview of the densely-packed human and other animal footprints, with a close view of P-01 (adult hominin), P-07 and P-09 (both produced by a young child).

**Figure 3:**
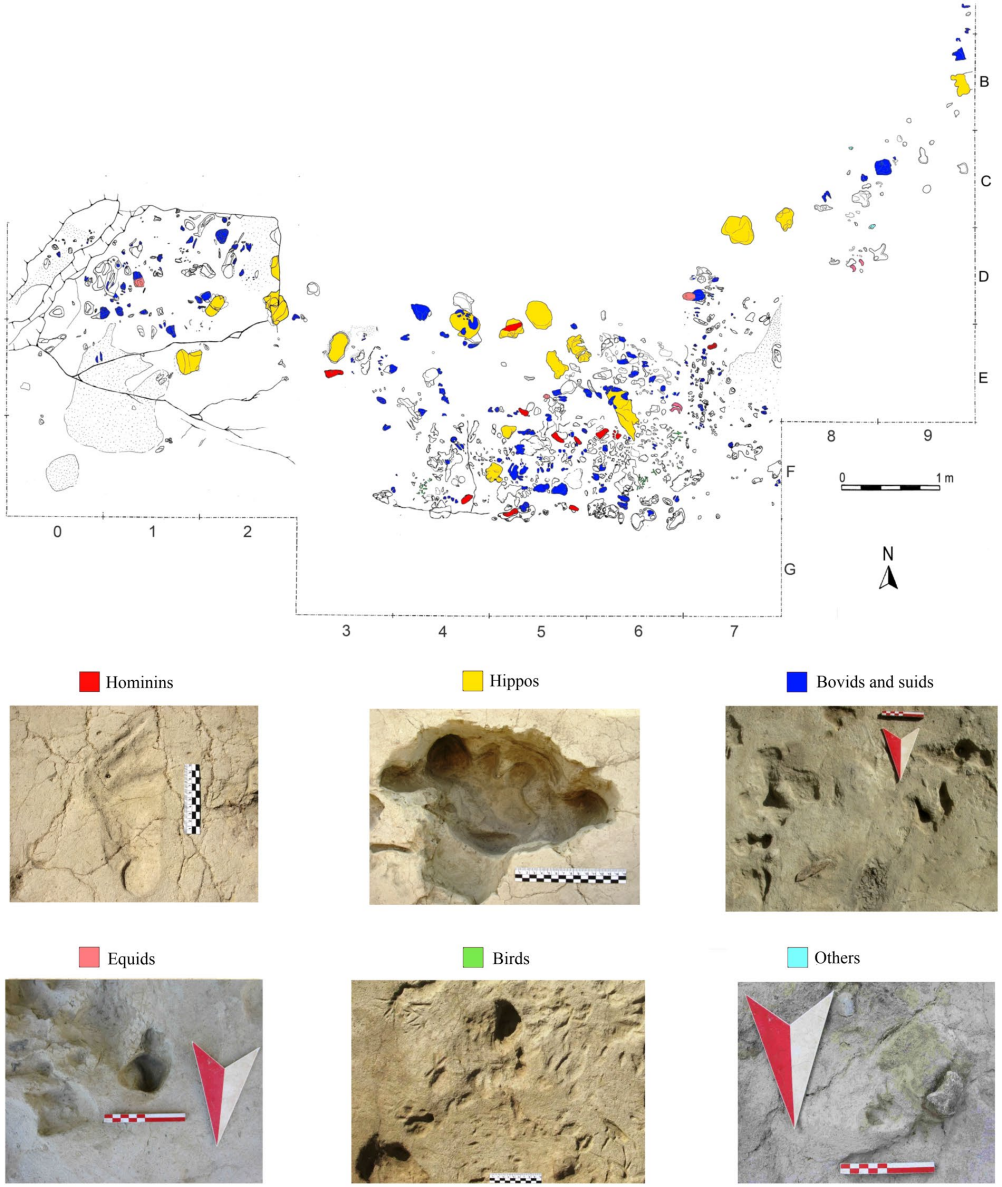
The ichnosurface of Gombore II-2. General planimetry of the excavated area (2013–2015) with colors referring to taxon attribution. Pictures of the best-preserved track(s) for each vertebrate group are provided: hominin (P-01, from square E3); hippo (sq. D2); bovid and possibly suid (sq. C8); equid (sq. E5); bird (sq. F6) and possibly a small carnivore (sq. C8).

The 2013–2015 excavations exposed a trampled sandy silt layer over ~35 m^2^ (Supplementary Figs S1-2). The upper surface of this layer slopes NW to SE and would have formed a shallow depression (~0.1 m deep) close to the southeastern limit of the excavated area. The trampled area stretches from east to west, extending beyond the NE and SE excavation walls. Previous excavations destroyed the northern portion of this layer. The footprints cluster in squares EF4–7, with a few additional tracks in the surrounding area. In densely packed areas, the tracks are so overprinted that few individual tracks preserve diagnostic features and trackways are absent. The Gombore II-2 ichnosurface is best described, therefore, as a ‘congregation’ site in which the tracks of various vertebrate species are grouped around a focal point^1^ (Figs 2 and 3). The focal point appears to be the shallow depression at the SE portion of the excavated area (Supplementary Fig. S1) which was probably occupied by a small water body.

During excavations thin, discrete sand lenses were recorded on the ichnosurface. This accumulating sand provided periodically fresh walking surfaces which implies that the animals visited the area regularly and in some cases tracks are compressed and/or superimposed through different layers (Supplementary Fig. S3). A range of hominin and other animal tracks are identified with reference to previous work in similar environments^12–14^. On this basis we recognize the following track-makers (Fig. 3): hominins, hippopotamus, bovids, equids, suids and birds. There is also a significant number of unidentifiable traces. The most frequent (>100 tracks) track-makers were large to small bodied bovids, potentially similar to gnu and gazelle in size (Fig. 3). In addition a total of 16 large hippo tracks (up to 0.33 m in diameter) were also identified. Bird prints of an unknown species are also common on the site. However, there are comparatively few suid or equid tracks and both lagomorphs and small carnivores were only tentatively identified.

**Figure 4:**
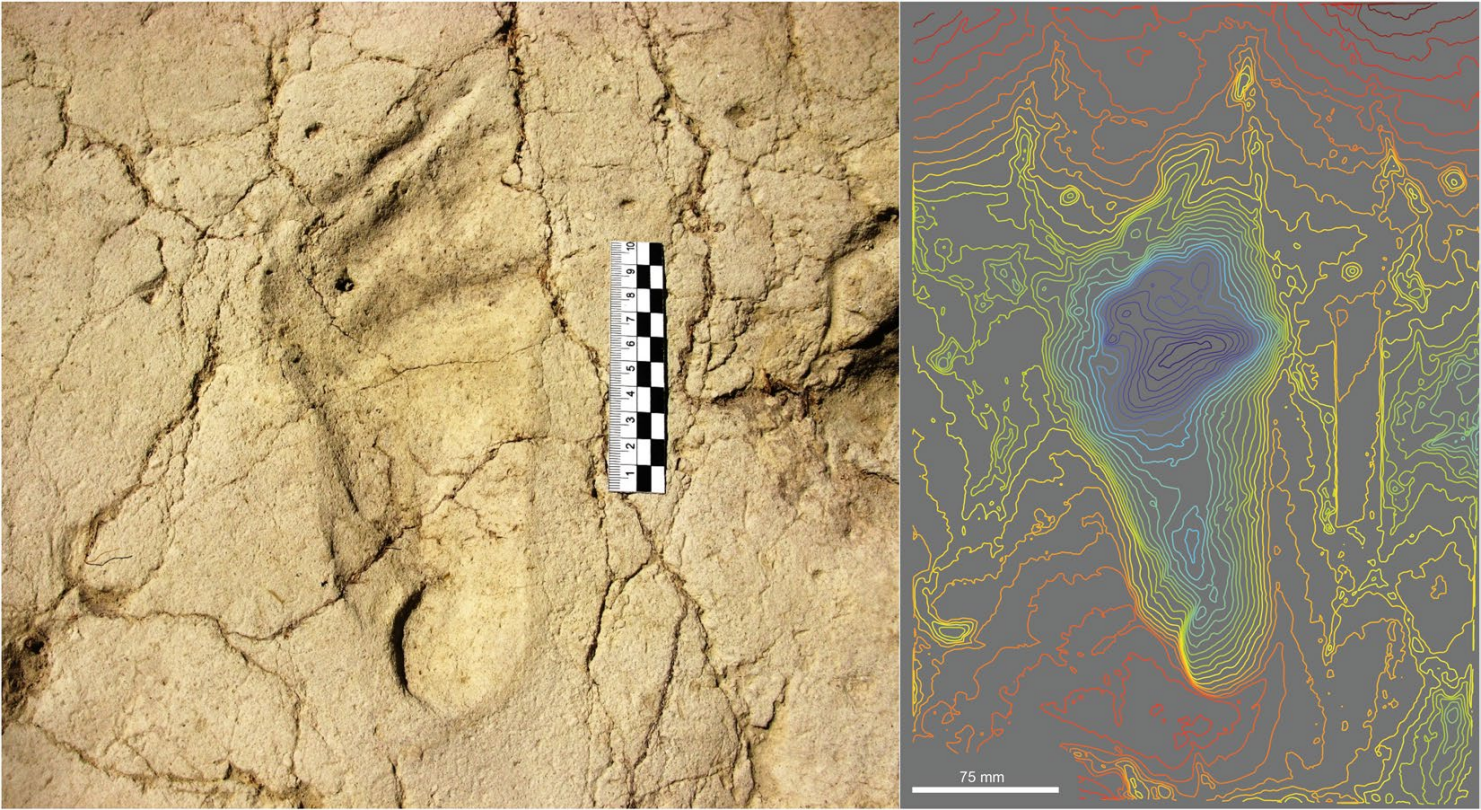
View and colour rendered model of the hominin track P-01, belonging to the left foot of an adult.

**Figure 5:**
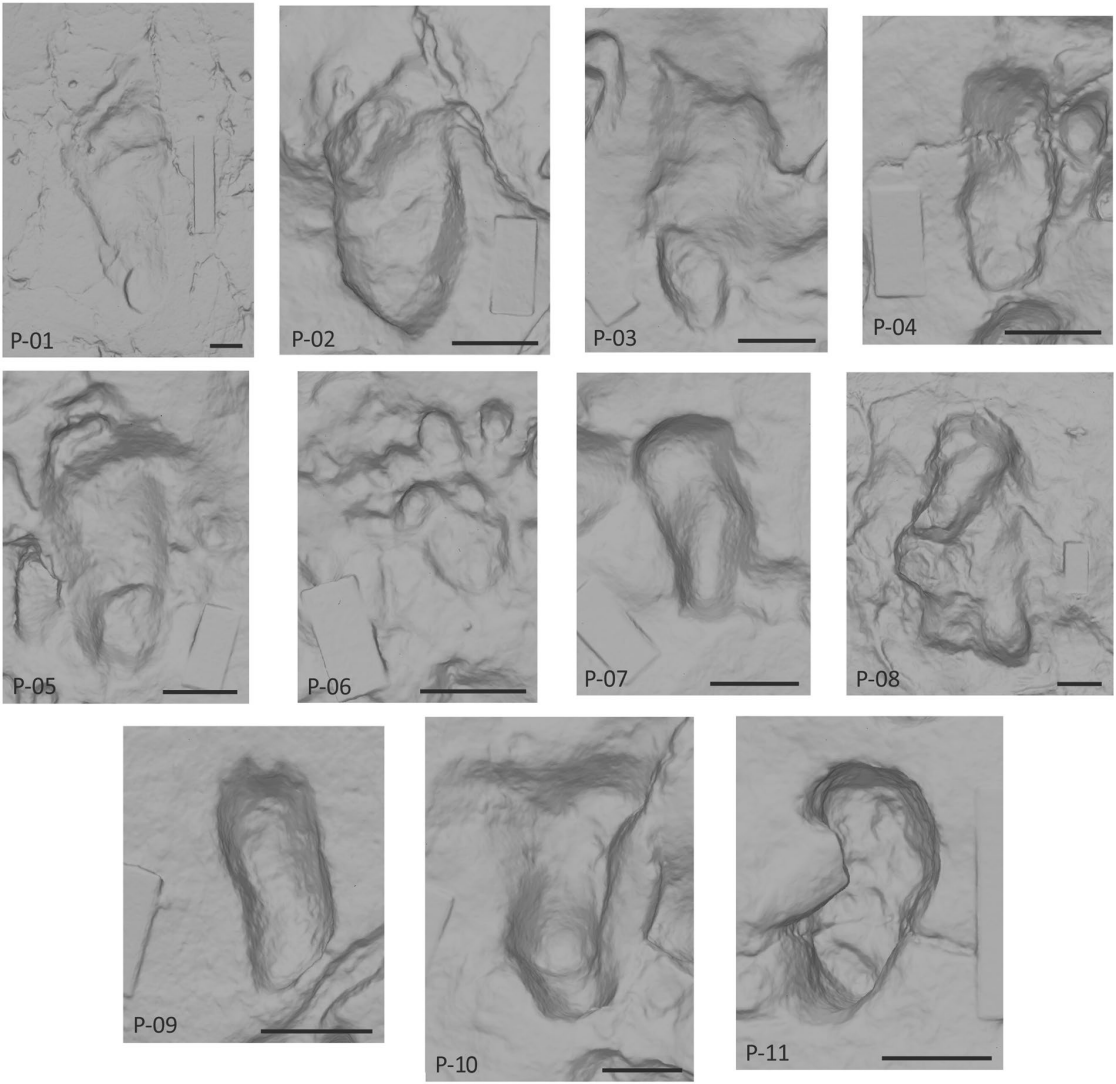
3D models of the hominin tracks. The scale bar is 50 mm (see Table 1 for dimensions). Models were made from oblique digital photographs in Agisoft (http://www.agisoft.com/).

Eleven possible human footprints were identified at the site (Figs 4 and 5; Table 1) with reference to other known footprint examples in East Africa in similar geological contexts^1,12–14^. These tracks have the characteristics proposed by Morse *et al*.^15^ as diagnostic of human tracks. The largest hominin track has clear and unambiguous anatomical definition (P-01: Fig. 4), with a rounded heel impression, visible digits and a drag line from the lesser toes. This track is comparable to other hominin tracks such as those identified at Ileret (Kenya)^12^. The smaller tracks pose a greater interpretative challenge however (Table 1). They have a variable morphology which has been impacted by adjacent tracks. This morphology ranges from simple kidney-shaped, flat-floored depressions (e.g., P-09, Fig. 5) to examples with potential toe impressions (e.g., P-05, Fig. 5). The two partial tracks in P-08 (Fig. 5), overlaying a hippo footprint, are instructive. The first track shows a vertical axial orientation in Fig. 5, and has a well-defined heel and a lateral foot margin that culminates in a partial first toe impression. Above this and orientated obliquely is a second small oval impression. Track P-09 shows drag marks associated with the removal of toes and P-06 shows a range of small toe impressions consistent with the multiple prodding of the substrate by a stationary individual. It is important to emphasize that the tracks are more consistent with someone standing in the mud rather than walking; hence the indistinct anatomy and multiple foot placements. In making an interpretation it is important to emphasize what they are not; they are not partial bovid tracks (i.e. one hemisphere) and they are morphologically inconsistent with equid, suid and small hippo tracks. Typical features of tracks made by modern young children (~12 months old) are shown in Supplementary Figure S4; note the tapering heel, the absence of a longitudinal medial arch and the relative proportions of the digits to the main body of the foot. Tracks of young children are also often associated with toe drag, consistent with a rolling or swinging gait.

**Table 1:** Track-maker stature and age estimates for tracks at Melka Kunture, Gombore II-2.

#	L (mm)	W (mm)	Estimated stature (m)	Estimated age (months) WHO Standards	Estimated age (years) Growth Curve	Estimated stride l (m)	Step width (m)
1	225.6	111.5	1.5		>Age-18		
2	138.3	72.6	0.92		Age 2		
3	156.3	67.8	1.04		Age-3		
4	113.0	47.6	0.74	6.5 (M) − 10.5 (F)	</=Age-1		
5	156.9	76.8	1.00	44.5 (M) − 47.5 (F)	Age-3	0.8	0.08
7	115.8	58.5	0.75	12 (M) − 13.5 (F)	</=Age-1		
8	274.6	144.9	1.83		>Age-18		
9	97.8	43.9	0.64	4.5 (M) − 5.5 (F)	</=Age-1		
10	107.1	34.0	0.72		Age-1		
11	115.2	55.4	0.77		Age 1–2		

Length is the maximum length along the long axis of the track, while width is the maximum width perpendicular to this axis. Stature is based on approximate relationship of foot length to stature of 15%. In the case of P-03 and P-05 it is possible to tentatively suggest that they may have been made by the same individual, hence the step width and stride length estimates. Track P-06 is not included here because it is incomplete and the product, we suggest, of multiple foot placement.

In terms of comparative material to assist with the interpretation children’s tracks were identified at Happisburgh (Norfolk) dating to 1 Ma (*Homo antecessor*)^16^. However no 3D data was collected at this site before it was destroyed and there are few published 2D images of the children’s tracks. Perhaps the best preserved, to date, children’s tracks are the Holocene tracks from Namibia^17^ (Supplementary Fig. S5). While these tracks are shallower than those at Gombore II-2 a simple landmark based comparison is possible. A total of twelve landmarks were placed on 20 tracks from Namibia and 7 from Gombore II-2 using DigTrace^18^ and exported. A simple Procrustes analysis on the landmark coordinates was then performed using PAST^19^. As illustrated in Figure S6 both sets of landmarks cluster within 95% confidence ellipses of one another despite the potential ontological differences between the track-makers^20–23^. This helps corroborate the interpretation of these tracks as being those of children.

We can also explore this further by attempting to infer the track-makers age. This is not without problems however, given that we do not have ontogenetic information for the inferred track-maker (*Homo heidelbergensis*). Here we used modern growth curves derived from those developed by WHO^24^, based on 2D foot lengths, following Ashton *et al*.^16^ to give a first approximation and to determine potential ages. Foot length growth curves for *Homo sapiens* are dependent on the individual’s sex as well as their ethnicity^25^ and crucially the levels of nutrition^26^. It is also worth noting that WHO growth curves are based on direct foot measurements, and not on 3D tracks made in soft and compliant substrates. Also in dealing with larger tracks, the potential for overlap between adolescents and adult females, given sexual dimorphism, needs to be considered^12,27^. To complicate this further the applicability of growth curves based on *Homo sapiens* to earlier hominin species is also potentially problematic. For example, Dean and Smith^28^ suggest that typical growth curve for early *Homo erectus* was more like that of a modern chimpanzees, than that of *Homo sapiens*, although unique to itself. The age estimates made here (Table 1) should be recorded as first approximations only. We used two complementary approaches. The first uses the foot length to stature relationship of Rutishauser^29^ followed by application of the WHO standards to provide an approximate age. The alternative method is based on a sample of 365 individuals mainly European, of which 149 were under 20 years old, for which 3D tracks are available and the track-makers’ ages are known (Supplementary Fig. S7). Dividing the data into yearly age classes and then using an adjusted percentile method of bootstrapping (N = 9999), confidence limits (95%) were estimated for each age class and used to infer ages from track lengths. Estimates based on the 3D growth curve place the track-maker of the smallest tracks (P-04 and P-09) equal to 12 months old, while the approach using the 2D WHO data gives ages that are younger potentially as young as 6 months old (Table 1). We don’t know whether *Homo heidelbergensis* infants were able to stand/walk at this early age or not, but in the case of *Homo sapiens* it is early. This potentially invalidates the interpretation of the smallest of the tracks as being hominin however it is worth noting that track P-09 has good anatomical definition with clear distal impressions associated with the withdrawal of toes and a defined heel (Fig. 5). On balance we believe that the most parsimonious explanation of the smaller hominin-like tracks is that they were made by very young children and taken as a whole there is evidence to support the interpretation of the hominin tracks as being from a mixed age assemblage.

Skeletal remains of *Homo heidelbergensis* have been recovered in the Middle Acheulean layer below the footprint-bearing surface^3^, which leads us to assign the hominin track-maker to this taxa. Even if other hominin taxa cannot be excluded completely, what is clear however is that the best-preserved adult track (P-01, Fig. 4), has a morphology that is consistent with other tracks made by *Homo*^1,12–14,17^.

## Archaeological Materials

As stated above, the Gombore track-makers, including hominins, walked around while sandy lenses accumulated across the area. The associated archaeological material is from the sandy lenses, just a few centimeters thick, and immediately above the top of the printed silty sand (Supplementary Fig. S3). Further evidence of direct association is provided by a basalt flake stuck in one of the hominin footprint (P-05, estimated age: 3 years), while a basalt core is superimposed on another track (P-02, estimated age: 2 years) (Supplementary Fig. S8).

Overall, fine- to very fine-grained sediments suggests a low-energy environment. This is in accordance with the state of preservation of the archaeological material. Lithic remains, including the fragile obsidian implements, show sharp edges which would not have survived any transport over a substantial distance. The isotropic distribution of the elongated pieces provides further evidence that the material was not re-oriented by water transport or other natural processes (Supplementary Fig. S9). We assume that the archaeological material is either *in situ* or only slightly displaced.

165 lithic implements and faunal remains were recorded during the new excavations from the sandy lenses and from the very top of the underlying ichnosurface (Supplementary Figs S9, 10, and Supplementary data), which since 2013 was otherwise left unexcavated to avoid destruction. During previous excavations, when the tracks were not yet recognized, 201 lithic and bone remains collected from immediately over and under this walking surface were recorded in bulk (see Supplementary data).

Within the lithic assemblage all stages of reduction sequences are recorded, further suggesting that knapping was taking place *in situ*. Most implements are flakes and debris, frequently made in obsidian. A few side-scrapers occur, but retouch rarely modifies the edges. Among the heavy duty tools, two basalt handaxes were discovered, one in 1995, the second one in 2014 re-deposited in the overlying ash flow (Supplementary Fig. S11). The technology, typology, raw material procurement and chronology are consistent with the Middle Acheulean, which is well documented at Melka Kunture^7^.

## The Faunal Remains

The associated faunal assemblage consists of 247 bone and tooth remains (see Supplementary data and Supplementary information, Table 1). Among the taxonomically determinable bones, *Hippopotamus* cf. *amphibius* dominates (n = 46). In addition, a first phalanx of *Equus* sp. was found. Most of the skeletal elements (n = 63) could not be attributed to Family/Order level, but only to animal size classes. Other bones (n = 137) could not even be attributed to size classes due to their high degree of fragmentation and surface concretion.

When possible the taxonomically indeterminable material was sorted according to size classes based on bone-thickness, taking into account the variety of taxa identified by Geraads^4,30^. Five size classes were defined. Size class 5 covers large-sized taxa such as elephants, hippopotamus and rhinoceros. With size class 4, the larger bovids (*Connochaetes*, *Pelorovis*) are covered. Size class 3 represents medium-sized bovids (such as *Damaliscus*, *Parmularius*) and Equids. With size class 2 the small bovids (such as *Gazella* and *Antidorcas*) are represented. Finally, size class 1 equates to mammals below the size of *Gazella*. All animal size classes were documented with the exception of size class 1. Hippos (animal size class 5) are almost exclusively represented by elements of the axial skeleton, (i.e., vertebrae, ribs and scapulae). Cranial elements are exclusively teeth, mainly canines and incisors. The humerus is almost always represented by shaft fragments, in contrast to the good preservation of femurs and tibiae, represented by complete bones or intact epiphyses with parts of the diaphysis attached. A minimum of three hippo individuals is identified on the basis of three left scapulae.

**Figure 6:**
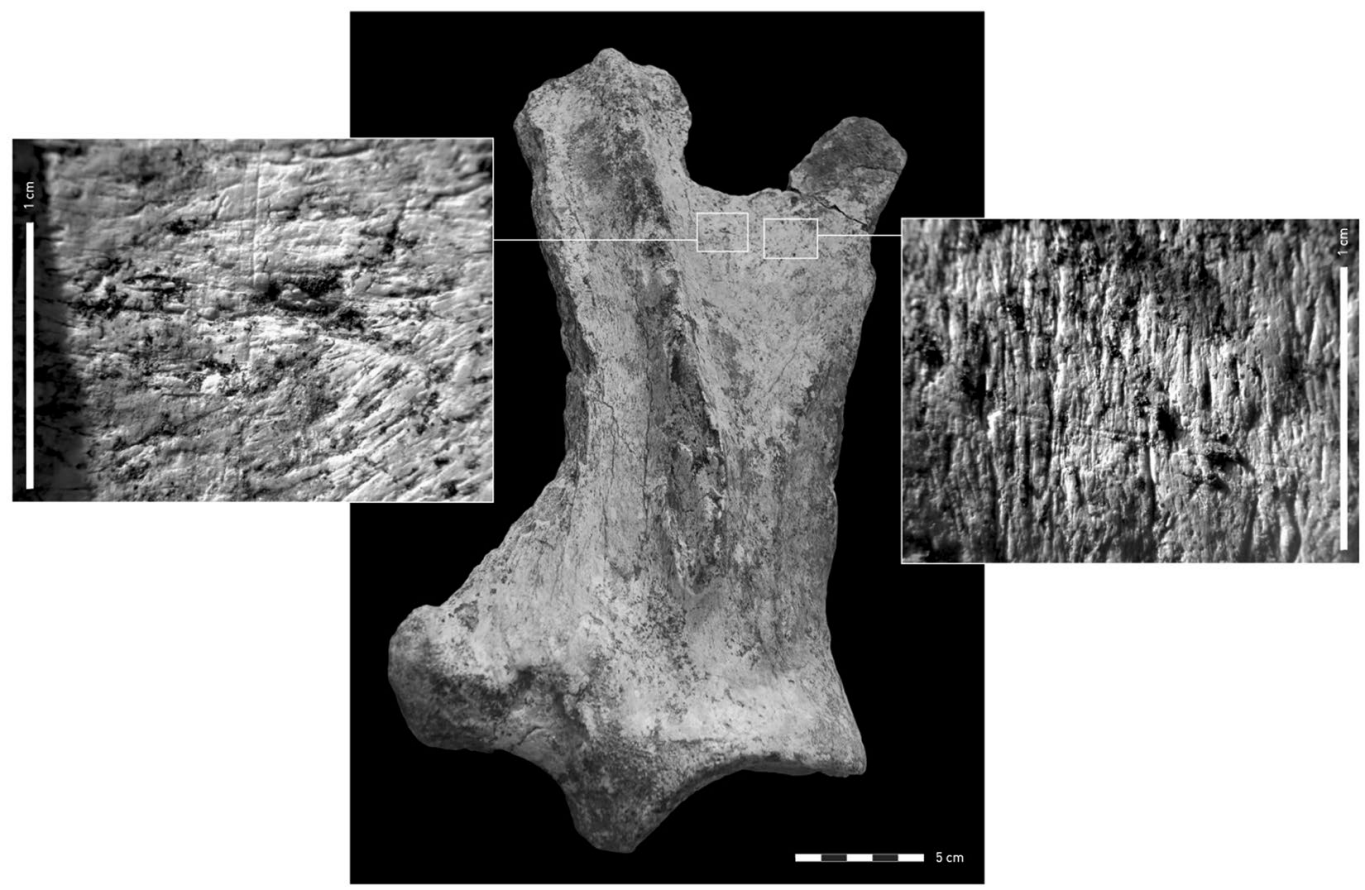
Scapula of *Hippopotamus* cf. *amphibius* with cut-marks made by lithic tools during butchering (*right insert*). *Left insert*: The cut-mark is superimposed by a carnivore tooth-mark, probably a scavenging hyena.

Analysis of fragmentation patterns for elements belonging to size class 5 (n = 71) allows recognizing an under-representation of spirally fractured long-bone shaft fragments compared to all other size classes. Characteristic however are elements displaying bending breaks (n = 9) indicating fresh bone breakage. Bending breaks almost exclusively characterize rib fragments that could be attributed to size class 5 animals. Bone flakes (n = 2) are equally present in the assemblage, resulting from long bone fracturing by either hominins or carnivores. Carnivore modification was particularly noted in bones of size class 5. Of the 71 skeletal elements attributed to *Hippopotamus* cf. *amphibius* and size class 5, 23 elements showed gnawing damage. This accounts for almost 32% of the assemblage. The general features of the gnawing damage^31^ suggest that the bones were subject to hyena scavenging. What is particularly striking are the homogeneous gnawing patterns evident for example on the scapulae which is best illustrated on scapula A-35 (Fig. 6). The cranial and caudal edges of the bone have been heavily gnawed, accompanied by tooth pits and tooth scratches. In addition the spine was almost removed. Microscopic analysis on two gnawed bone specimens revealed cut-marks made by stone-tools indicating defleshing and disarticulation of hippo carcasses by hominins. Among these specimens is the heavily gnawed scapula A-35, with cut-marks on its lateral surface. These traces were subsequently modified by a carnivore tooth mark, confirming that carnivores gained access to the carcass only after it was discarded by hominins (Fig. 6).

Overall, the faunal assemblage was affected by coatings of carbonate concretion (n = 79) which covered the bone surfaces to different degrees for all size classes (size class 2: n = 12, size class 3: n = 3, size class 4: n = 1, size class 5: n = 9, size class indet.: n = 27). This strongly supports the assumption that the burial milieu was comparable for bones of all size classes. To estimate exposure time between animal death and final burial, bone weathering stages were documented, following Behrensmeyer’s work^32^. For 157 faunal remains weathering stages could be determined, not all of which could be attributed to size  classes. The preservation of bone fragments of all size classes is mainly characterised by weathering stages 1 and 2 (n = 138). Weathering stages 3 und 4 were recorded for a further 8 specimens, representing size classes 2, 4 and 5. The documented weathering stages indicate that bones from differing size classes were buried relatively soon after the death of the animals. The assemblage composition was also considered according to the susceptibility to fluvial transport, expressed in the so-called “Voorhies groups”. Ribs, vertebrae, sternum and sacrum are subsumed under Group I as these bones are immediately moved by water. Scapulae, phalanges and ulnae belong to the intermediate Group I/II. Group II summarizes bones which are gradually removed, while the cranium and the mandible represent Group III, considered to represent a lag deposit^32^.

At Gombore II-2 skeletal elements of Groups I-III have been recorded, but most of the bones belong to Groups I and I/II. According to Behrensmeyer^33^, the presence of Group I bones indicates a non-fluvially winnowed assemblage. The degree of rounding was determinable only on a limited number of fragments (see Supplementary data) but it seems to correlate with bone fragment size and the degree of mineralization. Accordingly, we interpret small bone fragments as sediment components, as expected in a watering hole environment such as Gombore II-2.

## Discussion

Footprints do not last long at the surface when exposed to natural processes and anatomically diagnostic features may disappear within hours or days^13^. Exceptional preservation does happen if tracks are buried quickly in a low-energy environment and in these cases, they may record biological events happening just before they were infilled^9–11^. This contrasts with the archaeological record, which accumulates over time-lengths that are difficult to determine with precision. In the deeper and therefore earlier lithic- and fossil- bearing deposits at Gombore II-2 chronostratigraphic sequence disturbance is clear. For example, the 0.875 Ma Middle Acheulean layer was heavily reworked by water activity^7^. This contrasts with the archaeological evidence reported here, which shows little or no disturbance. What is clear from the sequence as a whole is that hominins repeatedly exploited the area ever since they returned during a favourable climatic episode of the Mid Pleistocene Transition, as suggested by Mussi *et al*.^5^. The interpretive challenge here is to reconcile different types of record; a footprint does not move and provides reliable evidence of *in situ* activity^10^, a snapshot into past life, while the archaeological record does not always provide this being more time integrated or averaged.

Gombore II-2 contains evidence of hominin tracks in association with a range of other animal tracks. The archaeological and faunal record provides evidence of the preparation of stone tools and carcass butchery. There are potential hominin tracks that due to their size may indicate the presence of children. The anatomy of these is not particularly distinct which we attribute to standing individuals rather than walking and that the ground was heavily trampled both before and after the specific track-making event.

At Gombore II-2, the track-bearing surface is slightly hollowed, and most impressions surround the depressed area which is assumed to have contained water (Fig. 3; Supplementary Fig. S1). The accumulated silt and sand lenses associated with the tracks point to periodic rises in the water level, depositing fine-grained sediment as small transgressive episodes that ‘refreshed’ the surface for new track making. The lack of any pedogenic processes or desiccation cracks on the ichnosurfaces points to a period of constant humidity and potentially therefore to a relatively short time span (<one season) prior to deposition of the over capping ash layer. A shallow pool would be expected to vary in water level substantially over a season and we do not see evidence of this. Moreover if the surface was exposed over weeks as opposed to hours/days we would expect a greater degree of trampling and degradation^13^. Specifically desiccated tracked areas around small water bodies like this tend to become brecciated very quickly when exposed for more than a couple of days. Blocks break from upstanding elements of tracks and infill the lower parts. Significantly no breccia was recovered as part of the track infill at this site. Furthermore, the ichnosurface is littered with archaeological and palaeontological material (lithics and bones) that do not appear to have been re-orientated by water flow. Bone preservation suggests that animal remains accumulated over a relatively short time interval, without significant displacement. Finally, lithic implements and bone fragments are impaled into some tracks and also overlying them. We suggest that the balance of probability therefore indicates that the footprints, knapping and butchery were all produced on site during a relatively short time interval and certainly within one season.

Perhaps the more controversial part of this story is the evidence for the presence of children. We argue that the interpretation of the small oval hominin-like tracks as being made by young children is consistent with the evidence. The challenge is that no single track is anatomically distinctive, however collectively one can recognize: (1) toe impressions; (2) nail scrapes associated with foot withdrawal; (3) rounded heels; and (4) peripheral outlines consistent with the correct anatomically position of toes and a medial longitudinal arch. This is supported by the quantitative comparison of the tracks with the children’s tracks from Namibia^17^ (Supplementary Fig. S6). At least two of the tracks are however very small in length, giving potential ages for the track-maker which (5–10 months; Table 1) which could challenge this interpretation. Unfortunately we do not know anything of the ontogeny  of *Homo heidlbergensis* infants. They may have been able to stand by 6 months and could have been placed upright in the mud to stand and watch. We simply don’t know. We do not believe that these two tracks however negate the interpretation of the hominin track assemblage as a whole of being of mixed-ages. Consequently we suggest tentatively that the implication here is that children were present during the butchery and tool preparation. As such the site may give insight into childhood around a million years ago. These results are consistent with those from other footprint sites where the presence of children’s tracks is relatively common^1^.

## Conclusions

The hominin tracks at Gombore II-2 add to the growing number of early track sites discovered in East Africa. The co-association of the hominin tracks with other activities, such as carcass butchery, and the production of the lithic tools is a novel feature when compared to other track sites identified to date. The interpretation is based on a powerful synergy of archaeology, geoarchaeology, zooarchaeology and ichnology data that points to methodological good practice which historically may not always have been followed. The butchered hippopotami and the association of a diverse fauna based on the ichnological record, confirm the richness of the area in terms of food availability. The presence of possible hominin tracks made by children as young as 1 year old, would imply that children had to tag along the adults in a mobile group and to learn first-hand information about hunting and butchering. Tool making, tool using and butchery behaviours are all inferred to be learnt skills^34,35^. We speculate that the presence of children’s tracks in direct stratigraphic association with such activities suggests that this learning occurred from a very young age.

The observations reported here complement those made at Ileret in Kenya^13,14^, where the preserved tracks, attributed to *Homo erectus*, tell a story of mobile all male hunting/foraging groups, with the females and juveniles, one assumes, left to other tasks in other locations. The implication of such a reconstruction is that adult foraging groups were perhaps the norm, with a division of labour, potentially along gender lines. Our observations of a later hominin species rather suggest mixed-age groups at least in certain contexts. Gombore II-2 illustrates the complimentary nature of ichnology and archaeology and its potential to allow a more comprehensive and inclusive approach to past life.

## Methods

In the nineties of the last century a French team, led by Chavaillon, established a general excavation grid at Gombore II-2, with each square meter identified by a progressive letter and number. We followed that grid and the related nomenclature as documented in the literature^5,7,8^. The deposit was removed progressively one thin layer at a time, following the excavation method known as “décapage horizontal”. Scalpels and small brushes were used to isolate and when needed, empty the footprints observed on the ground. Photogrammetric models of the tracks were constructed in Agisoft Photoscan (http://www.agisoft.com/). Analysis, visualization and contour outputs were executed in DigTrace (www.digtrace.co.uk). Zooarchaeological analyses focused on the identification of taxa, skeletal element representation and biotic and abiotic surface modifications. Bone surfaces were studied using a Dino-Lite PRO digital microscope with a magnification of up to 200x. All traces were registered per bone and recorded by anatomical position. Diagnostic criteria were used to identify hominin induced cut-marks^36^ and anthropogenic fractures^37,38^.

The archaeological collections, including the faunal remains, are kept at the National Museum of Addis Ababa. All the footprint data and scans from the excavation will be made available on the 3D sharing site http://morphosource.org.

Ethical approval for the modern analogue studies of children’s footprints prints is provided by Bournemouth University.

## Electronic supplementary material

Supplementary information
