# Supplementary material for: Archaeology and ichnology at Gombore II-2, Melka Kunture, Ethiopia: everyday life of a mixed-age hominin group 700,000 years ago
Source: Sci Rep. 2018 Feb 12;8:2815. doi: 10.1038/s41598-018-21158-7 (PMC5809588; doi:10.1038/s41598-018-21158-7)
Supplement: Supplementary file 1 — Supplementary information [file 41598_2018_21158_MOESM1_ESM.pdf]

Supplementary information for:

Archaeology and ichnology at Gombore II-2, Melka Kunture, Ethiopia: everyday life of a mixed-age hominin group 700,000 years ago

Flavio Altamura, Matthew R. Bennett, Kristiaan D'Août, Sabine Gaudzinski-Windheuser, Rita T. Melis, Sally C. Reynolds, Margherita Mussi

This file includes:

Supplementary data

Supplementary Figures 1-13

Supplementary Table 1

Supplementary data

The lithic industry from the trampled surface (2012-2015)

Between 2012 and 2015, 165 archaeological and palaeontological specimens were recorded from a 10 cm-thick sandy level (named level 4a) that caps the silty-sandy fossil ichnosurface (named level 4b). Level 4a was found only in the central and eastern part of the research area. Elsewhere the 0.7 Ma overlying volcanic ash directly capped the ichnosurface, with only residual sand filling the tracks.

We found 63 lithic remains within level 4a. Six were left *in situ* or were removed in a consolidated block of sediment. The average length of the remaining 57 lithics is just 3.5 cm. Only two are longer than 10 cm (specimens No. 706, 901).

As elsewhere at Melka Kunture, the preferred raw material is obsidian, accounting for almost half of the implements (28 finds). Other volcanic lithotypes were occasionally used (basalt= 15 specimens; ignimbrite= 11 specimens; flint and other volcanic rocks= 3 specimens).

The tools produced by *façonnage* or on a large support (heavy duty tools) are two choppers (No. 776, 921), a broken pebble (No. 740), two pebbles with percussion marks (No. 772, 780) and a rabot (No. 777). More than half of the remaining pieces

are flakes (38 specimens), often fragmented, mainly flaking *débitage*. This suggests that stone knapping was performed on site or in adjacent spots. Simple knapping methods (multidirectional and centripetal flaking) predominate.

Obsidian is the most common raw material for flakes (23 specimens). Basalt, welded ignimbrite and other volcanic rocks account for eight, five and two specimens, respectively. Obsidian flakes are small, never exceeding 4.3 cm, while those made from other rocks are up to 6.5 cm in length.

Three cores were recorded. The biggest one is a basalt core (No. 901, see Fig. S8), with two convex and tangent flaking surfaces, that was centripetally flaked. The two other cores, in welded ignimbrite, are residues of multi-directionally exploited blocks (No. 723-724).

Three flakes were modified into side-scrapers (No. 715, 729, 951), and one turned into a scaled piece (No. 768), while another one bears a notch (No. 720). Two more flakes have marginal retouch (No. 742, 957).

Level 4b was not removed to avoid damaging the fossil ichnosurface. However, 12 lithic artifacts and 16 faunal remains were either recovered from the ichnosurface or found outcropping in the area of last century excavations, where a limited extension

of layer 4b was still in full view. The average length of lithic artifacts in this level is 7.2 cm, which is more than double the average length of the finds from level 4a. A single retouched flake is on obsidian. Two cores, a flake, a side-scraper, a rabot, as well as broken pebbles and pebbles with percussion marks are all made using a variety of basalts.

The lithic industry of the ichnosurface from previous excavations

In 1974, a 7 m^2 test trench was dug. It was just 20 cm deep, leading to the discovery of faunal remains and of 22 lithic implements. In 1993 and 1995, 26 m^2 more were excavated. After our own observations and analysis, during past research the subdivision into levels 4a and 4b had not been observed or taken into account, and the remains had been lumped together. The over one hundred bone fragments and 48 lithic tools of the time[4] are from level 4 without any further subdivision. Out of a grand total of 70 lithic implements we were able to relocate only 36 finds from the original excavation, which are kept at the Melka Kunture and ARCCH storehouse in Addis Ababa, Ethiopia. Instead, we made use of inventories in the Mission’s archive that had also been used in a preliminary study of the lithic industry[4]. Basalt and trachy-basalt are the most common raw materials. A core, four flakes and two retouched tools were made in obsidian, while a small piece of flake *débitage* documents the occasional use of jasper. There are 21 heavy duty tools (15 pebble with fractures and percussion marks, four choppers, two rabots) and 11 implements

obtained by *façonnage*: 6 polyhedrons, 4 bolas and the only handaxe so far directly recovered from level 4, a single basalt implement. The nine cores are multidirectional ones. 14 flakes were found; 13 more flakes were modified by retouch and occasionally turned into side-scrapers, or other tools types.

Fossil preservation

The bones from Gombore II-2 showed different stages of fossil preservation. Some of the bones only survived with their trabecular parts, though this was only documented for small fragments. Other bones had disintegrated into a powdery substance though many of these highly fragmented bone splinters still had the outer surface preserved. These elements could not be considered for further analysis.

The faunal assemblage was severely affected by coatings of concretion which covered the bone surfaces to different degrees. In the case of the bone remains retrieved during the early stage of research, It appeared that concretions had been partly removed due to some bone treatment after excavation. Therefore only the presence/absence of these surface coverings was documented.

Rounding

Stages of rounding where defined for the current study, with Stage 0, the absence of rounding up to Stage 5, completely rolled bones. Stages of rounding (Fig. S12)

could only be documented for a limited amount of faunal material (n=181). Differences in the degree of rounding were observed for different size classes, suggesting a connection between rounding and bone fragment size. This is well illustrated by a comparison of bones belonging to Size Class 5 (n= 68) that show a homogeneous rounding pattern whereas bones from Size Class 2 (n=16) primarily display rounding according to Stage 4, i.e. bones affected by heavy rounding with the original shape of the bone still recognizable (Fig. S13).

Moreover, a correlation between the degree of bone mineralization and the degree of bone rounding was noted, in that bones staging heavy rounding were often also heavily mineralized. Due to the regular presence of concretions which affected bone surface preservation this observation could not be further quantified.

A low-energy environment with only calm sediment deposition that preserved under a volcanic ash characterizes the geological outcrop at Gombore II-2. The isotropic spatial distribution pattern of lithics and faunal remains underlines low-energy deposition. Indications signaling material intake into the site by high energetic processes due to e.g. water transport are lacking in the sedimentary sequence and the skeletal element representation for Hippo does not display the selected composition expected for faunal assemblages transported by water. For the faunal assemblage of Gombore II-2 it can thus be excluded that the degree of bone-rounding as well as the degree of mineralization are indicative of the relative time

between the death of an organism and its final burial. This is also underlined by the observation that bones coated with concretions sometimes displayed different degrees of rounding. This strongly suggests that diagenetic processes probably connected to the deposition of the volcanic ash on the ichnosurface acted *in-situ* upon the autochthonous faunal assemblage.

Supplementary Figures

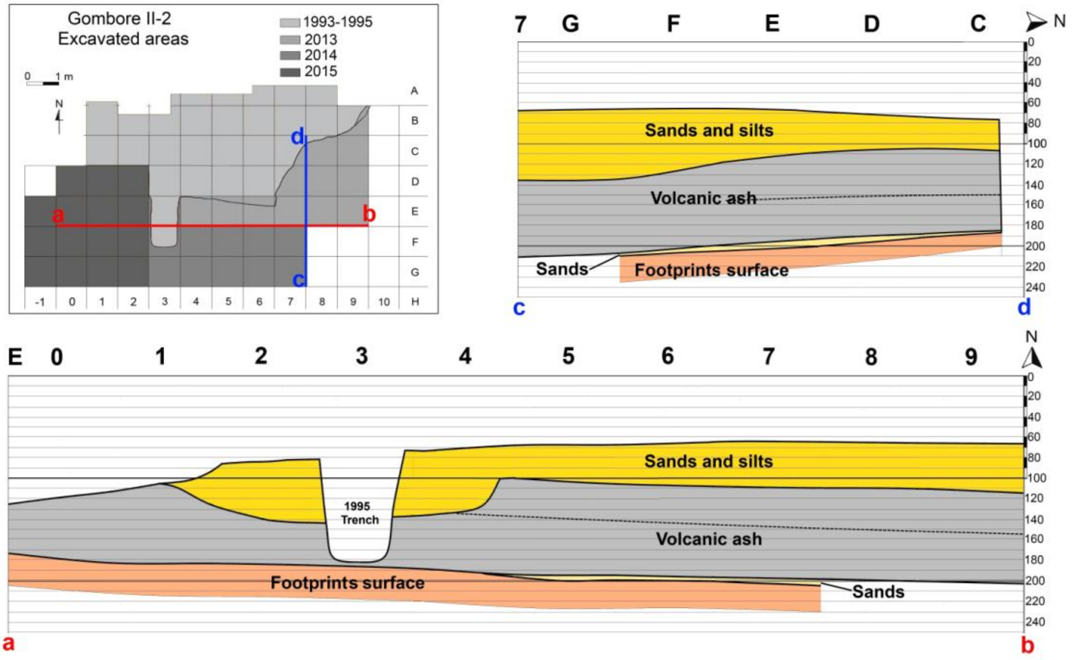

Supplementary Figure 1 | Gombore II-2 excavation. General planimetry with grid and partition by excavation years, with stratigraphic profiles (a-b and c-d). In the E-W section (a-b, square rows 5-7), note that the slightly depressed surface of the footprint-bearing layer is filled by sand, suggesting that a small pond had been in existence.

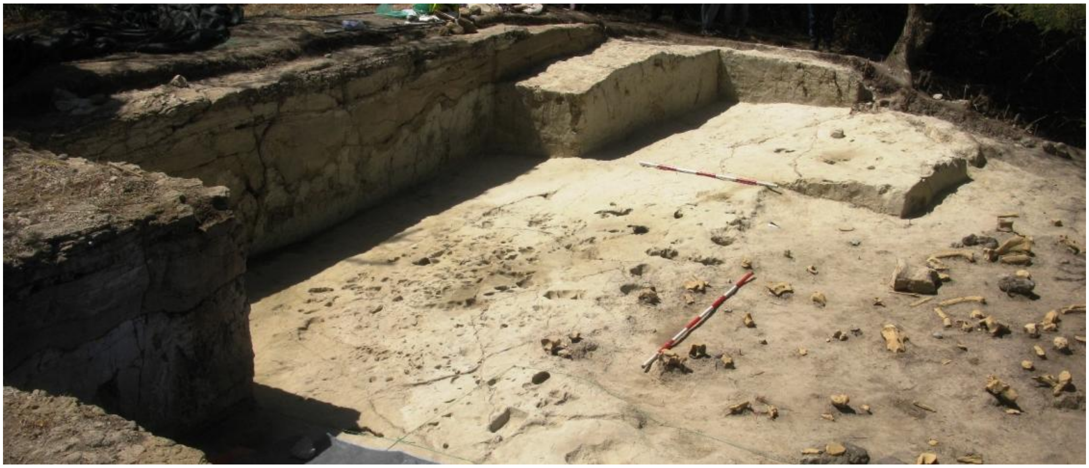

Supplementary Figure 2 | Gombore II-2 excavations. General view from N-E of the exposed footprint surface (2013-2015). On the right, the area excavated in 1993 and 1995, with resin casts of bones and lithic materials repositioned in 2001.

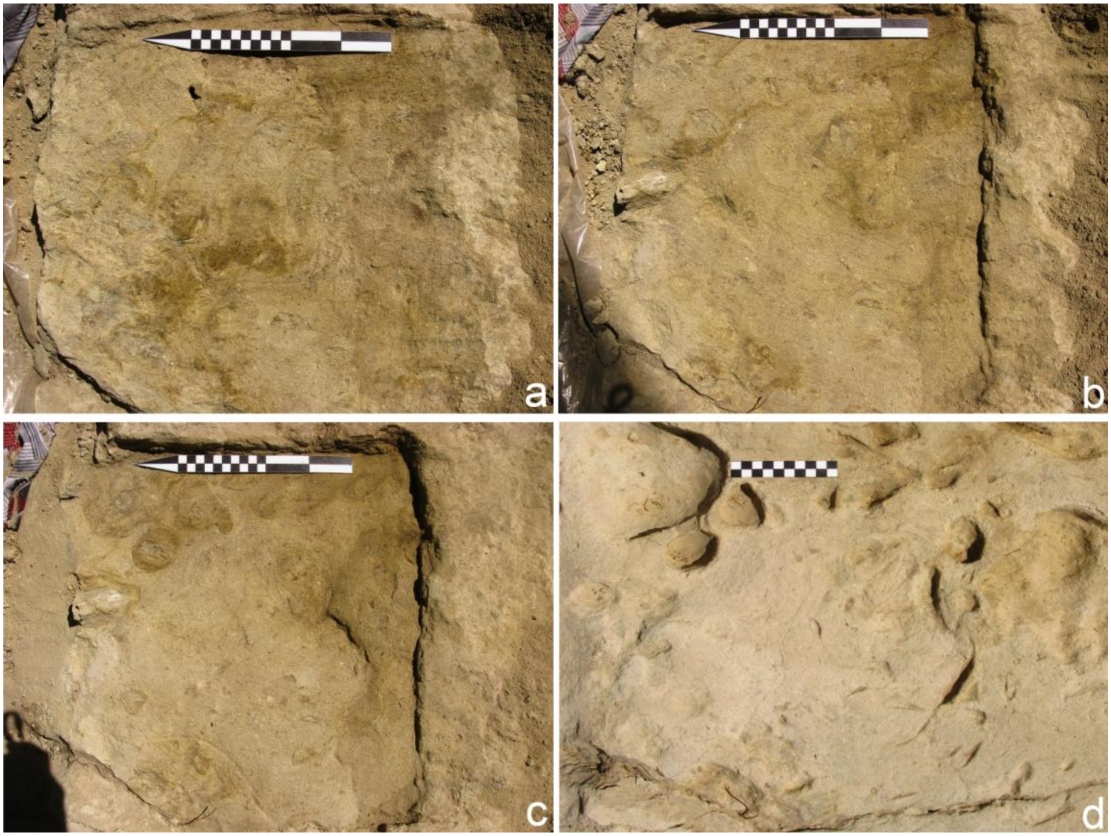

Supplementary Figure 3 | Gombore II-2, excavation of the sands overlying the ichnosurface (NW sector of square F5). The pictures were taken each 1 cm while progressively excavating, ending with the complete exposition of the ichnosurface. Bioturbations are evidenced by discrete spots of sands of slightly different colour. Hominins and animals were walking on the sands, often sinking in the underlying plastic sandy silt. This implies that the finds from the sands are related and contemporaneous with the ichnosurface (e.g. see bone No. 907 on the left corner of the pictures b, c), i.e. they were deposited during a relatively short period of time while the walking surface formed.

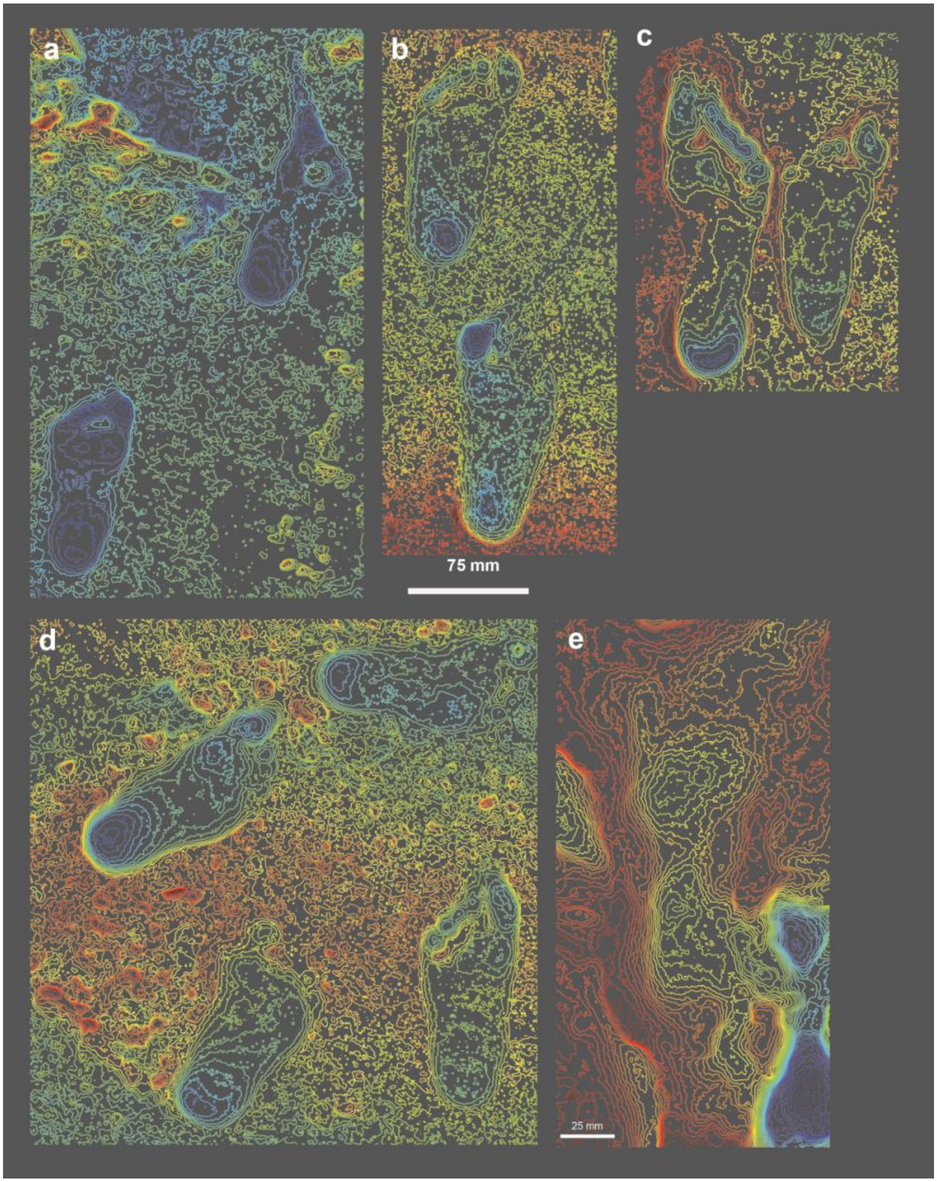

Supplementary Figure 4 | Colour rendered models of modern and ancient human tracks used for comparison. Tracks a, b and d were experimentally obtained by children between the ages of 9 and 18 months walking in a sand tray. Tracks c were left by a young adult (left) and a one-year old child (right) walking in a sand tray. This formed part of an experimental project at Bournemouth University (Foot Lab, 2014) with ethical permission courtesy of Bournemouth University. The assistance of Talbot Campus Nursery is acknowledged.

Track e belongs to a sub-adult and is from the lower footprint surface of Bennett et al.[12]. The track is approximately153 mm long. Contour intervals are 1 mm. Three-D models were using a Konica-Minolta VI900 optical laser scanner and processed in DigTrace (www.digtrace.co.uk).

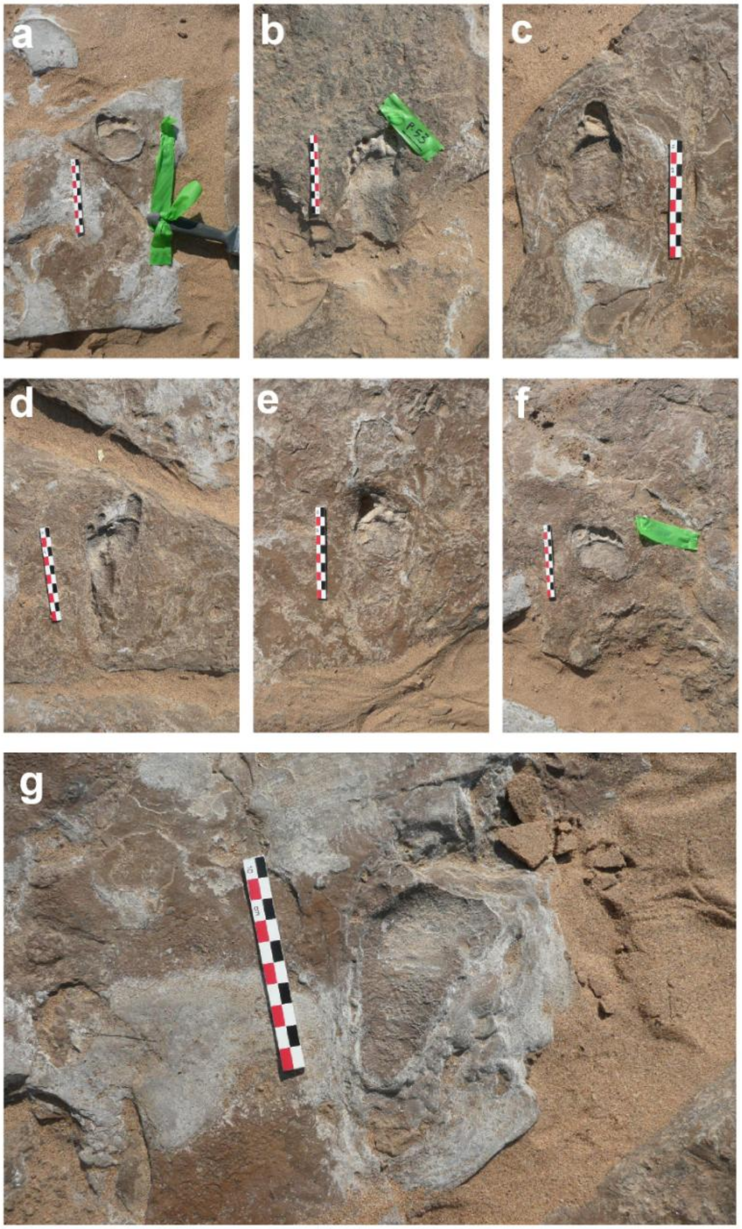

Supplementary Figure 5 a-g | A range of tracks from Namibia[17] showing typical morphological features of small children including variable indentation, absence of longitudinal medial arch, strong toe impressions. Age estimates for these tracks provide values between 3 and 5 years.

.

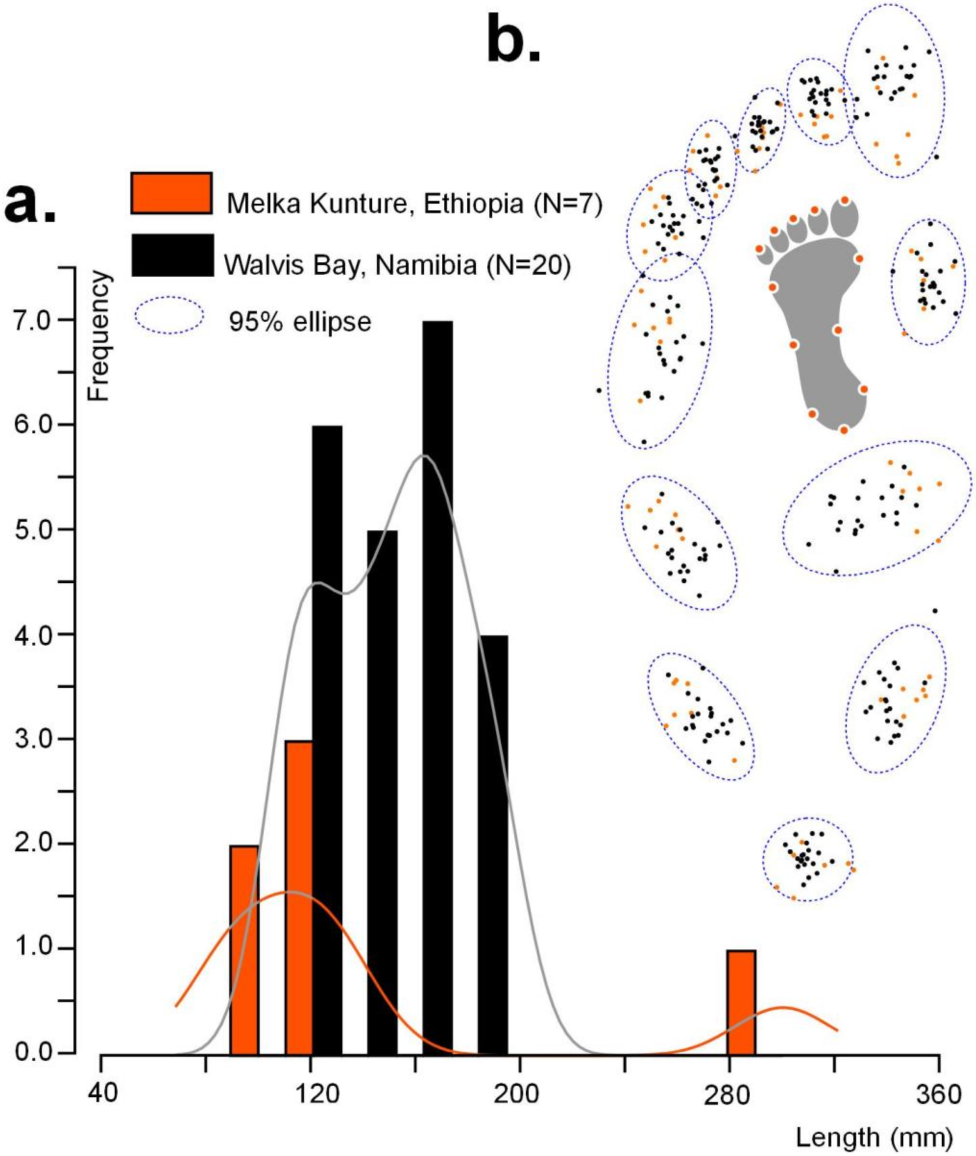

Supplementary Figure 6 | Landmark based analysis of the Gombore II-2 footprints compared to those from Namibia[17] (Fig. S5). a. Foot length comparison between the two sites, with kernel density curves. b. Landmark comparison of the two samples with 95% confidence ellipses indicated. Grey inset shows the landmark positions.

.

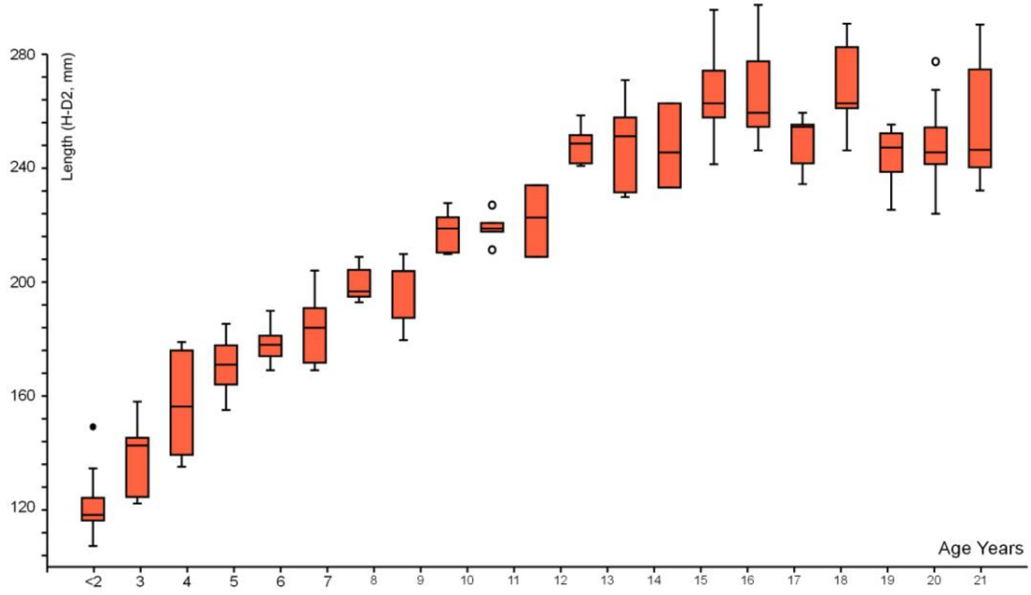

Supplementary Figure 7 | Growth curve for foot length (Heel to D2) for 3D tracks made either in a sand tray or on Bournemouth Beach (2015). Based on a sample 149 individuals under 21 years old of a mix of ethnic groups, although predominantly Caucasian. Confidence intervals are based on bootstrapping with 95% confidence limits.

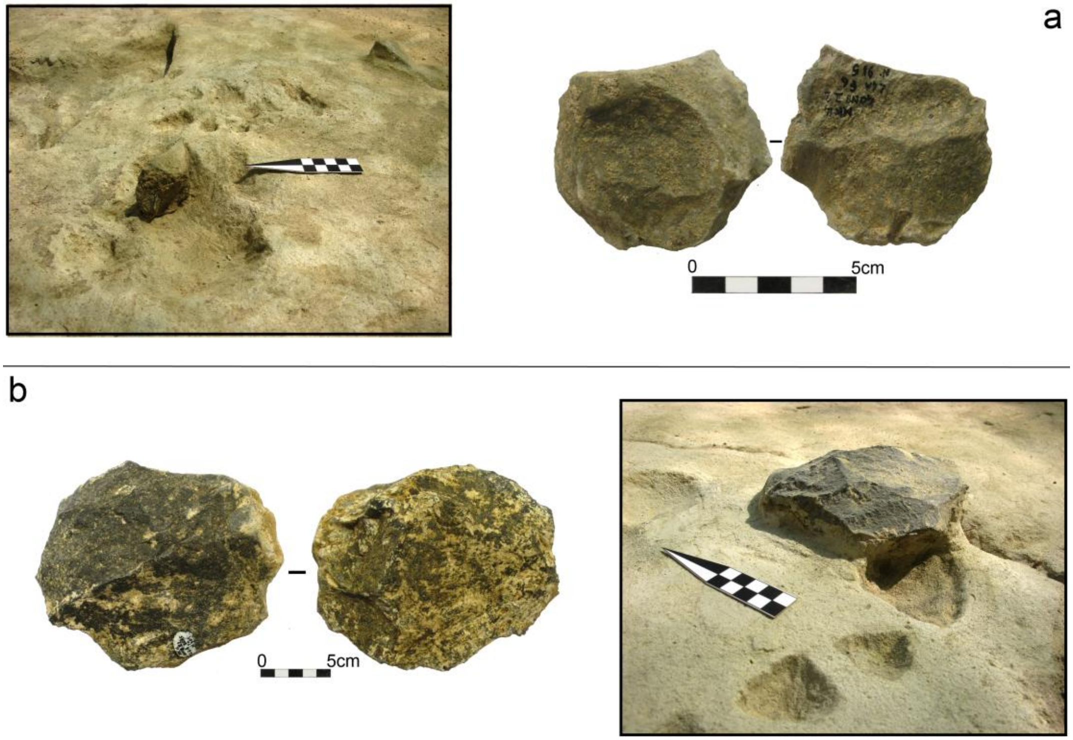

Supplementary Figure 8 | Lithic industry directly associated with human footprints on the Gombore II-2 ichnosurface. a) A basalt flake (No. 915) vertically stuck in the sandy infill of P-05; b) A basalt core (No. 901) laying on the top of the infill of footprint P-02. In the picture the infill is already partially removed by the excavation process.

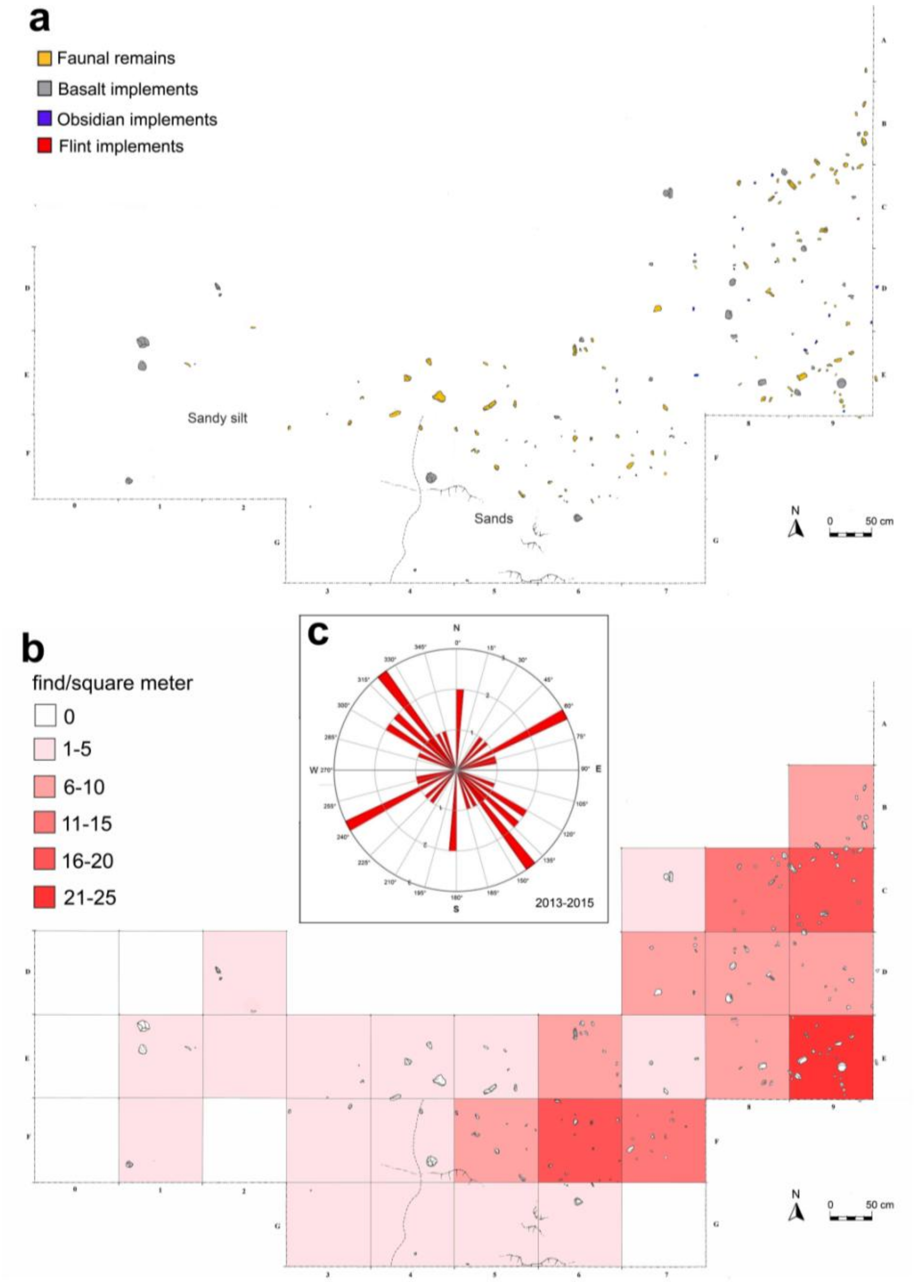

Supplementary Figure 9 | Gombore II-2, 2013-2015 excavations. Plan of the excavated area with the location of the archaeological finds recovered in the sands covering the trampled sandy silt, and on the top of the ichnosurface itself (a). Plan with the excavation grid evidenced and a colour-based characterization of the number of finds in each square meter (b). The material from the sieve was added to the total; in squares G5-7 the sands were only partially removed. Rose diagram of the orientation patterns of the elongated finds (length >5 cm, at least twice the width: n.= 20) from the sands overlying the ichnosurface (c). The diagram shows an isotropic pattern of orientation consistent with a low-energy environment.

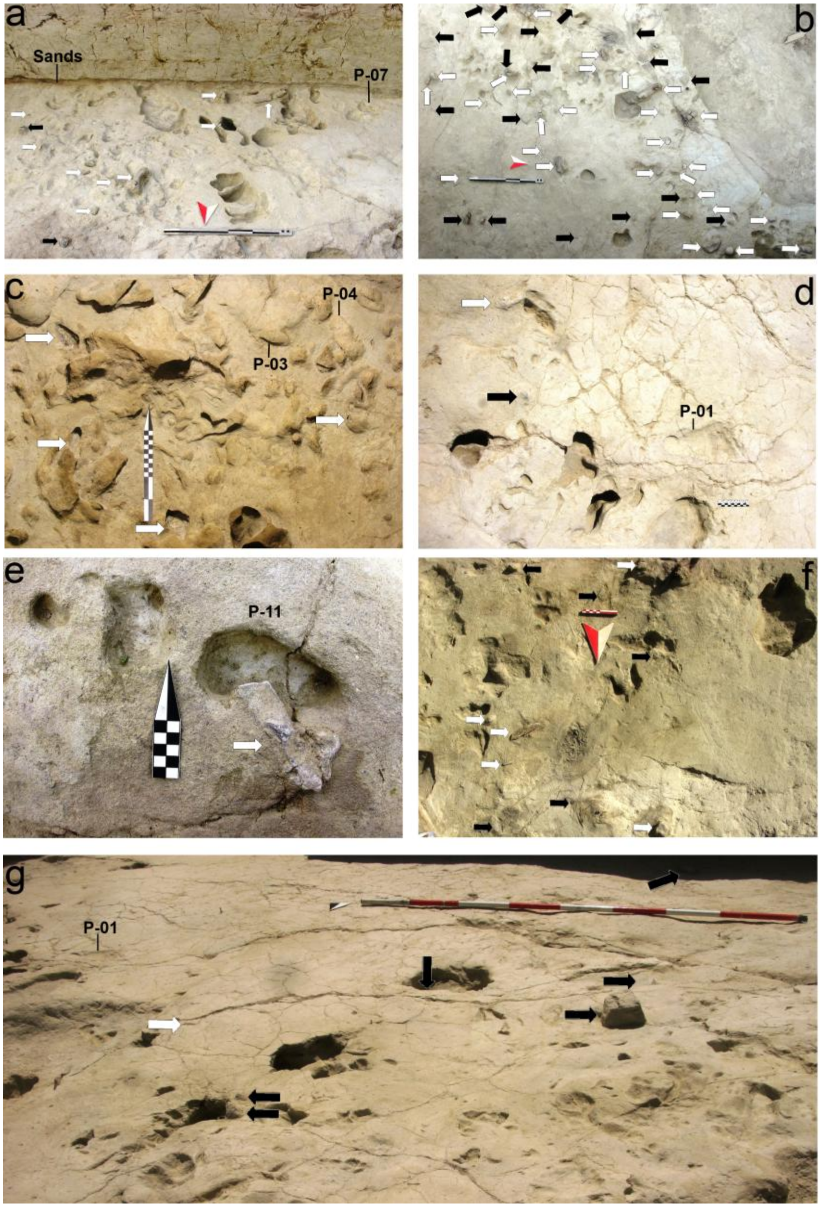

Supplementary Figure 10 | Gombore II-2, details of the associated archaeological remains and footprints. Faunal remains (white arrow) and lithic implements (black arrow) laying on the walking surface, i.e. stuck on the top of the trampled sandy silt or included at the base of the overlying sands: a) co-association of lithic and fossils on the ichnosurface exposed in squares E5-6 in 2013; note the child track P-07 and the sands, few centimeters thick, which are sandwiched between the ichnosurface and the ash deposit and can be seen along the nearby standing wall; b) archaeological finds and footprints in squares BC8-9 (2013); c) faunal remains in square F5, laying on the surface close to P-03 and P-04 (2014); d) the hominin track P-01 in square E3, close to more tracks and to a bone fragment and an obsidian flake (2014); e) animal bone stuck into the infill of the child footprint P-11, (square F5, 2015); f) co-association of archaeological remains and animal footprints in square C8 (2013); g) slanted view of the ichnosurface in squares DEF0-3 (2015); note hominin footprint P-01 and a big basalt core outcropping from the trampled surface at a distance of a couple of meters. More animal footprints and archaeological remains appear in the picture (scale with 20 cm sections).

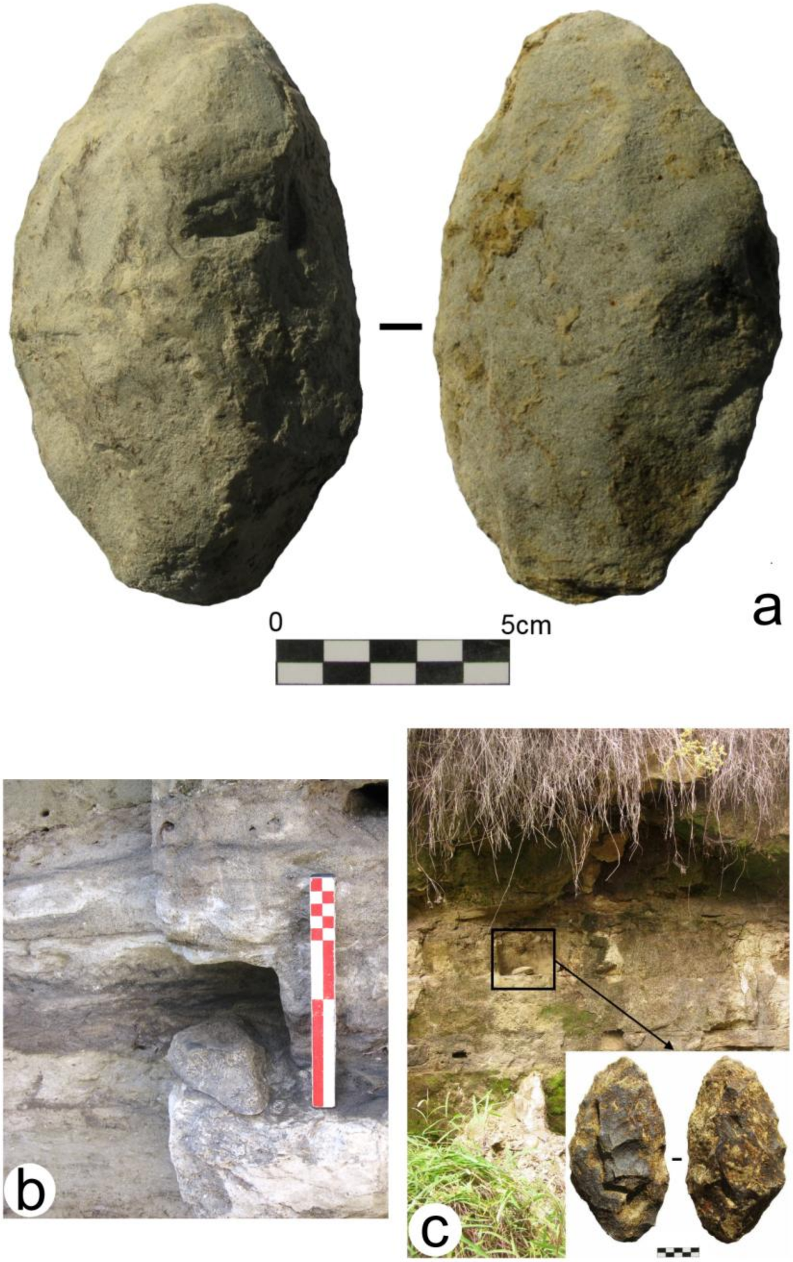

Supplementary Figure 11 | Handaxes from Gombore II-2 excavations. a) Basalt handaxe (No. 873) discovered in 2014 at Gombore II-2 within the ash which caps the ichnosurface. In 1995, another basalt handaxe (MK95, Gom. II-2, No. 175), not illustrated here, was discovered within level 4 in square D3. b-c) Two handaxes found at Gombore X, a natural section located ~30 m south of Gombore II-2. They were included in sandy and silty-sandy layers also covered by the 0.7 Ma volcanic ash, i.e. in the same stratigraphic position as the ichnosurface.

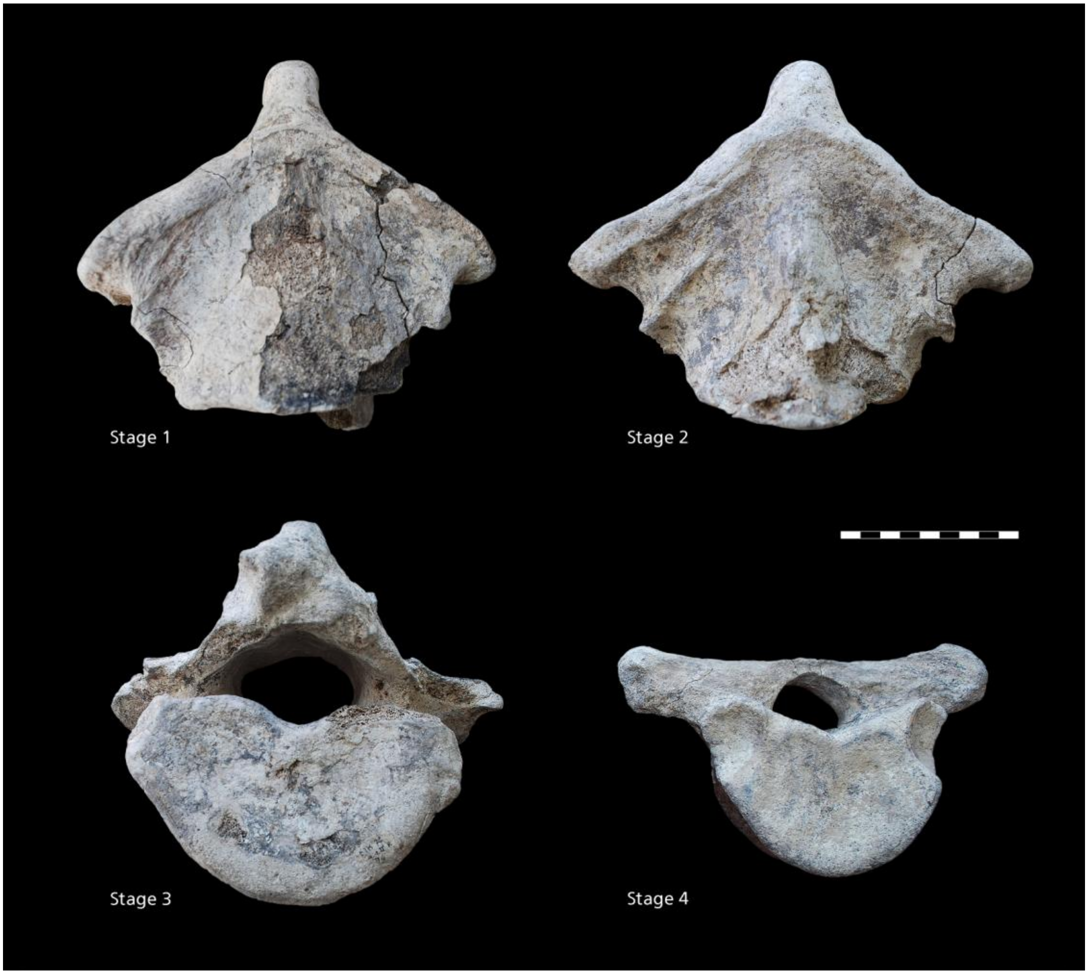

Supplementary Figure 12 | Gombore II-2. Stages of rounding illustrated by Hippo vertebrae. Stage 1: sporadically slight rounding, Stage 2: sporadically rounding, with protuberances visible, Stage 3: rounding, with protuberances hardly visible, Stage 4: Complete rounding of the bone that preserved its general shape.

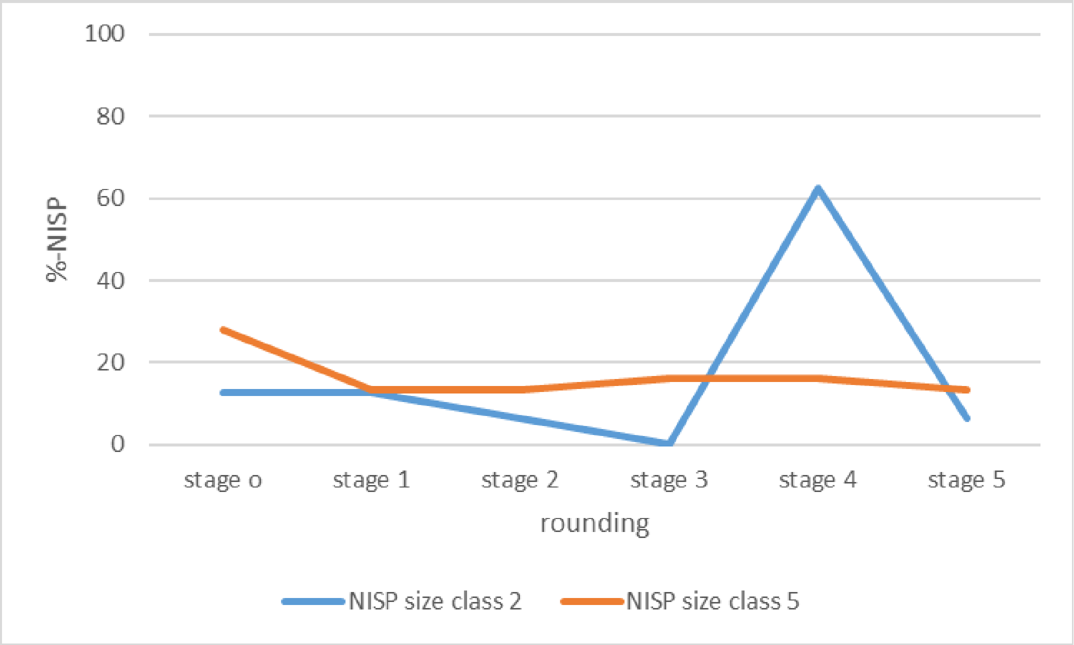

Supplementary Figure 13 | Gombore II-2. Stages of rounding documented for bones belonging to Size Classes 2 and 5.

Supplementary Table

Supplementary Table 1 | Composition of the faunal assemblage from Gombore II-2, layer 4 (grand total from the 1974, 1993, 1995, and 2013-2015 excavations). NISP = number of individual specimens per taxon.

		NISP
Hippopotamus cf. *amphibius*		46
	cranial	7
	axial	33
	longbones	5
	autopodium	1
Equus sp.		1
	autopodium	1
size-class 2		26
size-class 3		7
size-class 4		5
size-class 5		25
	cranial	1
	axial	3
	longbones	15
	autopodium	1
	indet.	5
indet.		137
traces of carnivore modification		44
traces of hominin modification		3
